# Solid-State NMR Study of Hydrochars Produced from Hydrothermal Carbonization of Poultry Litter

**DOI:** 10.1021/acsomega.4c02876

**Published:** 2024-11-04

**Authors:** Mariana C. Santoro, Bashir M. Ghanim, Witold Kwapinski, James J. Leahy, Jair C. C. Freitas

**Affiliations:** † Laboratory of Carbon and Ceramic Materials, Department of Physics, Federal University of Espírito Santo (UFES), Av. Fernando Ferrari 514, 29075-910 Vitória, Espírito Santo, Brazil; ‡ Department of Chemistry, The Higher Institute of Medical and Technical Sciences, Alzahra, 00000 Tripoli, Libya; § Department of Chemical Sciences, Bernal Institute, University of Limerick, V94 T9PX Limerick, Ireland

## Abstract

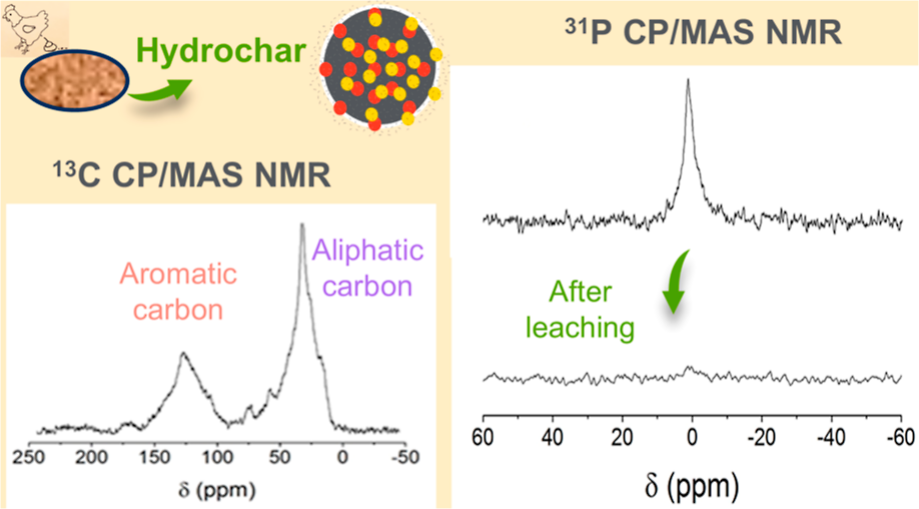

Poultry litter (PL) hydrochars obtained at different temperatures and charring times were characterized by solid-state ^1^H, ^13^C and ^31^P nuclear magnetic resonance (NMR) spectroscopy. ^13^C NMR spectra obtained with cross polarization (CP) and magic-angle spinning evidenced the chemical and structural changes suffered by PL during its transformation into hydrochar; these changes were particularly dependent on the production temperature rather than the residence time. The hydrochars were essentially composed of aromatic and alkyl domains at the temperature of 250 °C. ^31^P NMR observations were conducted using single-pulse excitation (SPE) and CP sequences to distinguish between phosphorus far from protons and protonated phosphate species. Results showed that water-soluble phosphorus was the only form detected in hydrochars through the CP sequence. In contrast, the stable phosphorus species formed during hydrothermal carbonization (HTC) exhibited broad signals, detected exclusively using the SPE sequence. This indicates that unprotonated orthophosphates were the dominant form. These NMR results offer a deeper understanding of hydrochar formation from PL, shedding light on the chemical and structural changes caused by the HTC process at the atomic scale.

## 1 Introduction

Agriculture and livestock production are growing sectors of the economy due to the growing global population demand for increased food production. Developing alternatives for these sectors to become more productive and sustainable is crucial, considering the problems food production might face in the following years through climate change, decreased agricultural land areas and phosphorus scarcity. Unlike nitrogen, the primary source of phosphorus is derived from phosphate rock, a nonrenewable resource estimated to become scarcer and costlier in the coming decades. Continuous application of fertilizers containing phosphorus, nitrogen, and potassium is vital to sustain high crop yields, and investing in P recovery technologies from secondary sources is critical for future food security.^1,2^

The recovery of phosphates from biomass, wastewater, and manures provides sustainable alternatives to traditional phosphate procurement methods, such as rock mining. However, the application of soil organic amendments, like manure and poultry litter (PL), can exceed the nutrient uptake of crops, resulting in the accumulation of fertilizers in soils and subsequent leaching. Consequently, while phosphorus scarcity is a future concern, the present issue of phosphorus leakage from soils and wastewater into surface water leads to eutrophication. This process, driven primarily by phosphorus surplus, causes harmful algal blooms in freshwater ecosystems.^3^

PL, a dryer feedstock, is easier to transport and combust compared to other types of litter and slurry. It contains bedding materials, excreta, decomposed manure, spilt feed, feathers, and water, creating a heterogeneous material.^4^ While it enhances soil fertility in traditional farming, intensive poultry farming can lead to regional manure accumulation and overapplication, causing nutrient leaching, odors, greenhouse gas emissions, pathogen growth, and soil acidification.^5^ Environmentally, thermal treatment of PL is preferable to direct land application.^5^

Biochar, derived from pyrolysis, and hydrochar, from hydrothermal carbonization (HTC), have garnered significant attention as sustainable approaches for carbon sequestration, soil enhancement, greenhouse gas mitigation, and waste management in recent years.^6−9^ HTC is an interesting option for wet materials, such as microalgae, sewage sludge and manures, once it does not require a drying stage before the thermal treatment. It is performed at lower temperatures than pyrolysis to produce chars with a significant level of recalcitrant carbon, typically rich in nutrients, particularly nitrogen and phosphorus.^10^ The solubility and precipitation of phosphates under hydrothermal conditions are primarily influenced by reaction temperature, residence time and initial pH. Furthermore, the quantities of minerals and metal cations present in each precursor significantly impact phosphorus speciation during the reaction.^11,12^

The carbon sequestration potential of biochars and hydrochars can be attributed to their recalcitrant nature, which depends on the degree of aromaticity and aromatic condensation.^13^ Solid-state nuclear magnetic resonance (NMR) spectroscopy, a nondestructive method, provides detailed information on molecular species in heterogeneous matrices. It is beneficial for studying chars due to its sensitivity to the local chemical environment of the investigated nucleus. Solid-state ^13^C NMR can assess the structural configuration of carbonized materials, indicating their stability,^14−16^ while solid-state ^31^P NMR evaluates the chemical environment of phosphorus.^17−20^ The commonly used sequences in solid-state NMR involve the combination of magic angle spinning (MAS) with either single-pulse excitation (SPE), with the direct polarization of the analyzed nucleus, or cross-polarization (CP), which depends on the magnetization transfer rate from ^1^H to the nuclei in question. The indirect excitation of ^13^C or ^31^P nuclei through dipolar coupling with protons amplifies the signal magnitude compared to direct polarization by SPE, especially when these nuclei are in proximity to protons within environments exhibiting limited molecular mobility and devoid of interactions with paramagnetic or ferromagnetic centers.^21,22^

The chemical shifts of orthophosphates in solid-state ^31^P NMR can be shifted from those reported in solution NMR due to hydration effects, cation complexation and adsorption on minerals.^23,24^ The degree of covalence on the O–P–O bond and the electron donor capacity of hydrating water molecules can cause an upfield shift on the NMR spectrum due to the increased shielding of the P nucleus. On the other hand, replacing hydrogen from the P–O–H phosphate by a cation can reduce the electron density around the P nucleus, deshielding it and leading to downfield shifts.^23,25^ Although phosphate species are not easily assigned in solid-state ^31^P NMR, this technique can provide information to complement chemical extraction results or be combined with NMR of other probe nuclei present in the material.^24,25^

The ^13^C NMR analysis of PL biochars obtained after various pyrolysis conditions has given insight into the carbonization process of this precursor. Cimò et al.^26^ used ^13^C CP/MAS NMR spectroscopy and thermogravimetric analysis to characterize poultry manure biochars produced under varied temperatures and residence times. Their findings underscore the substantial impact of temperature on the aromatization of poultry manure, overshadowing the influence of residence time. In another study, developed by Jiang et al.,^19^ the authors applied ^13^C and ^31^P MAS NMR spectroscopy, using SPE and CP sequences, to characterize raw PL and PL-derived biochars prepared at different temperatures. At 600 °C, the ^13^C NMR spectra revealed the conversion of organic matter into stable aromatic structures. Concurrently, ^31^P NMR results showed the decomposition of phytates, and the formation of Ca and Mg phosphates for temperatures above 500 °C. In both studies, solid-state NMR spectroscopy proved invaluable for characterizing char, offering insights into converting PL to biochar with targeted environmental applications.

Solid-state ^1^H NMR is less frequently used to characterize solids due to the large homogeneous broadening that affects the resolution of the ^1^H NMR spectra. In these spectra, the homonuclear dipole–dipole interaction is the principal reason for the line width. However, ^1^H NMR may offer the possibility to analyze the proton mobility of solid samples and indicate the transformation of organic groups.^27,28^ Further information on ^1^H dynamics can be obtained with the 2D ^1^H–^13^C wide-line separation (WISE) technique, which involves the detection of the ^1^H signal via indirect detection on the ^13^C channel. A CP step is performed first, which depends on the heteronuclear dipolar coupling for magnetization transfer from ^1^H to ^13^C, and then the ^1^H detection is filtered via high resolution on the ^13^C channel.^29^ This method allows the separation of linewidths of resonances due to protons bonded or close to ^13^C nuclei from different functional groups.^29^

Ghanim et al.^30−32^ conducted detailed studies characterizing hydrochars derived from PL. In their previous studies, the authors reported the influence of treatment temperature, residence time, and initial pH on: (i) the hydrochar yields and the chemical properties of the prepared samples, carrying out proximate and ultimate analysis besides cellulose and lignin quantification and (ii) the speciation of phosphorus in the synthesized hydrochars, using chemical extractions according to the SMT protocol^33^ and quantifying the contents of phosphorus and other nutrients present in each fraction. Despite being extensively studied, understanding the chemical and structural changes governing carbonization and phosphorus speciation during hydrothermal processes remains incomplete.

The present work aims to employ a comprehensive suite of solid-state ^1^H, ^13^C, and ^31^P NMR techniques to further investigate the PL and PL-derived hydrochar samples, which were previously studied by Ghanim et al.^30−32^ The ^13^C CP/MAS spectra were recorded to understand better the aromatization process occurring during HTC of PL, while ^31^P CP/MAS and SPE/MAS NMR experiments were conducted to elucidate further the protonation and bonding state of the phosphate groups. These results were also complemented by 2D ^1^H–^13^C WISE experiments, which provided information on the nature and mobility of the hydrogen-containing groups responsible for the polarization transfer to the ^13^C nuclei in the CP experiments.

The NMR results obtained in the present work were thoroughly compared with the results presented by Ghanim et al.^30−32^ The main goal of this work is to use solid-state NMR as a tool for the further characterization of the same samples, aiming to understand the structural and chemical changes caused by the HTC process at the atomic scale, by probing the changes in the chemical environment of the chosen nuclei (^1^H, ^13^C, ^31^P). The quantitative results can be correlated to the information obtained by solid-state NMR spectroscopy, using the multinuclear approach described, providing new insight into the HTC process and the chemical properties of the produced hydrochars.

## 2 Results and Discussion

### 2.1 Effects of HTC Reaction Conditions on the Carbon Matter of PL

**Figure 1:**
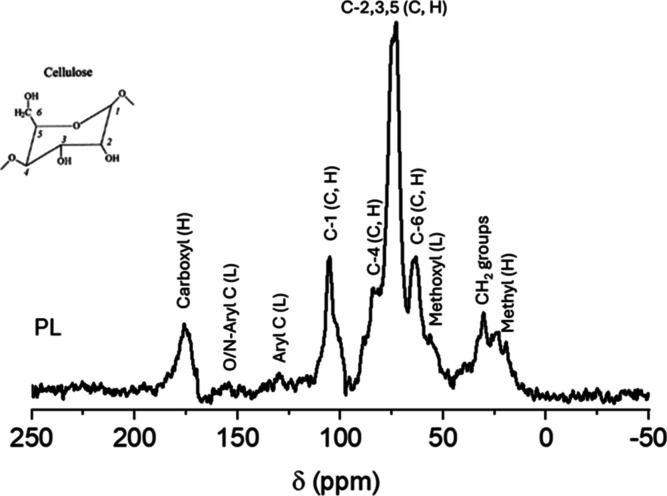
^13^C CP/MAS NMR spectrum of PL. The letters C, H and L indicate the signals associated with cellulose, hemicellulose and lignin, respectively. The inset shows the numbering scheme used to identify the atoms in the anhydroglucose repeating unit of cellulose.

The ^13^C NMR investigation was conducted to evaluate the effect of the HTC on the chemical shifts and lineshapes of the signals present in the spectra of the PL-derived hydrochars, focusing on the effect of temperature and residence time on chemical/structural changes in hydrochars. The ^13^C CP/MAS NMR spectrum recorded for the PL sample is shown in Figure 1. The signals between 0 and 50 ppm are related to alkyl carbons present in this material. The peak around 30 ppm is attributed to poly methylene (–CH_2_–) chains; the upfield shoulder (26–10 ppm) is typical of terminal methyl C and methylene C in close vicinity to electrophilic groups such as carboxyl C; methyl groups of hemicellulose are also known to contribute with a signal close to 20 ppm.^26,34^ The resonance at 30 ppm (which was the most intense in this spectral region) is likely associated with long-chain fatty acids present in poultry manure, which give rise to characteristic peaks with chemical shifts around 30 ppm (–CH_2_– groups), 130 ppm (olefinic/aromatic C) and 172 ppm (carboxyl groups).^26^ The other prominent peaks in the PL spectrum correspond to the presence of carbohydrates resonating between 50 and 110 ppm, the *O*-alkyl region, with chemical shifts typical of cellulose (and also of hemicellulose): the strongest peak around 72 ppm is due to carbons C-2, C-3, and C-5, whereas peaks at 105, 84 and 64 ppm are respectively related to carbons C-1, C-4, and C-6 (see atom numbering in the inset of Figure 1). The upfield shoulder of the C-1 signal can be assigned to amorphous cellulose or hemicellulose C-1.

The signal at 56 ppm is due to methoxyl groups present in lignin, a further important biopolymer present in PL, likely coming from the bedding material; weak signals observed in the aromatic region of the spectrum (roughly from 115 to 160 ppm) are also associated with lignin. Signals of ethers and alcohol C inside chains of lignin units, hemicellulose and tannins add to the region commonly associated with carbohydrate signals (*O*-alkyl C). The somewhat broad resonance at 173 ppm contains contributions due to carboxyl C from hemicellulose and organic acids.^35−37^

**Figure 2:**
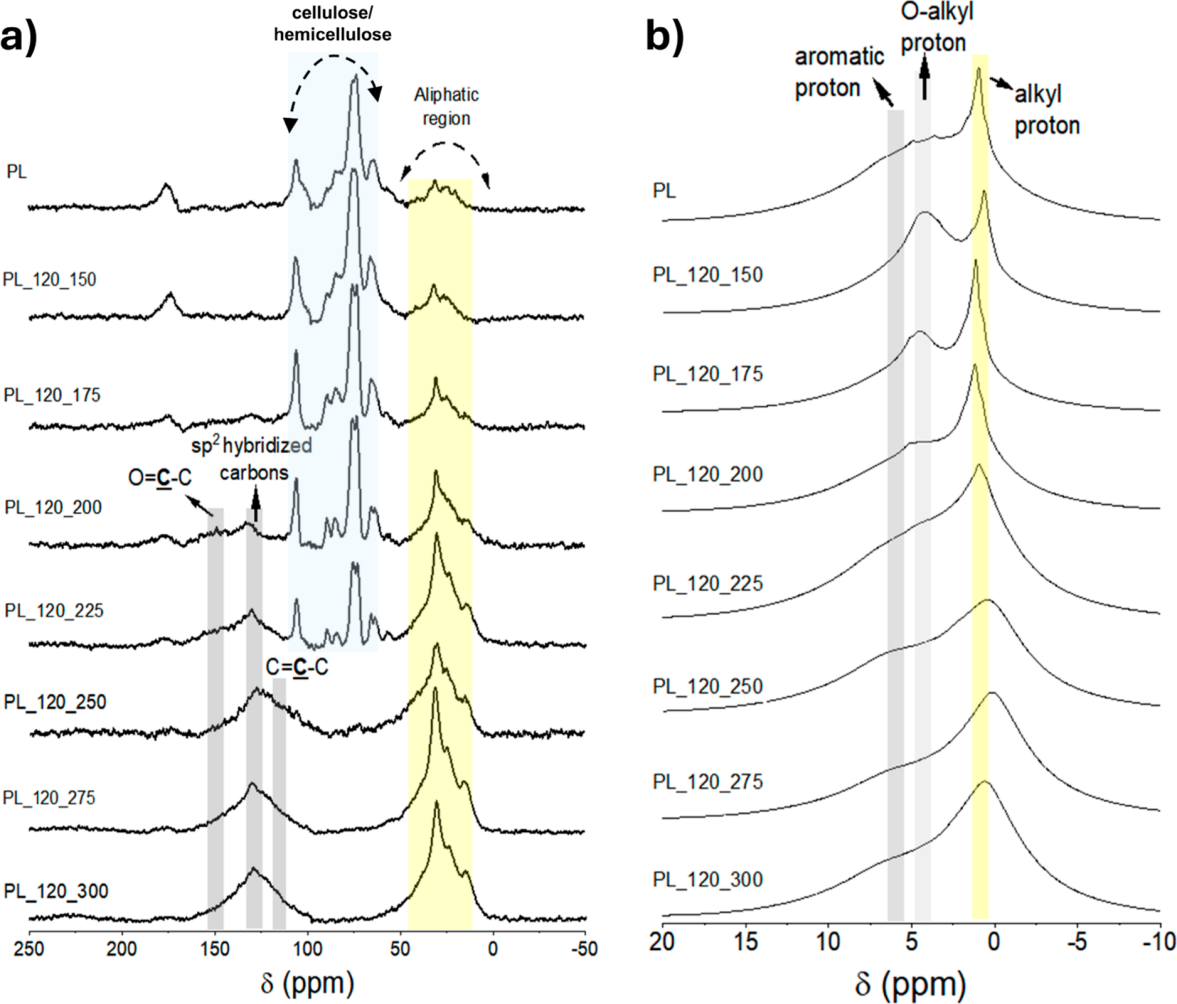
NMR spectra of raw PL and hydrochars produced at increasing treatment temperatures and 120 min of residence time: (a) ^13^C CP/MAS NMR spectra and (b) ^1^H MAS NMR spectra.

**Figure 3:**
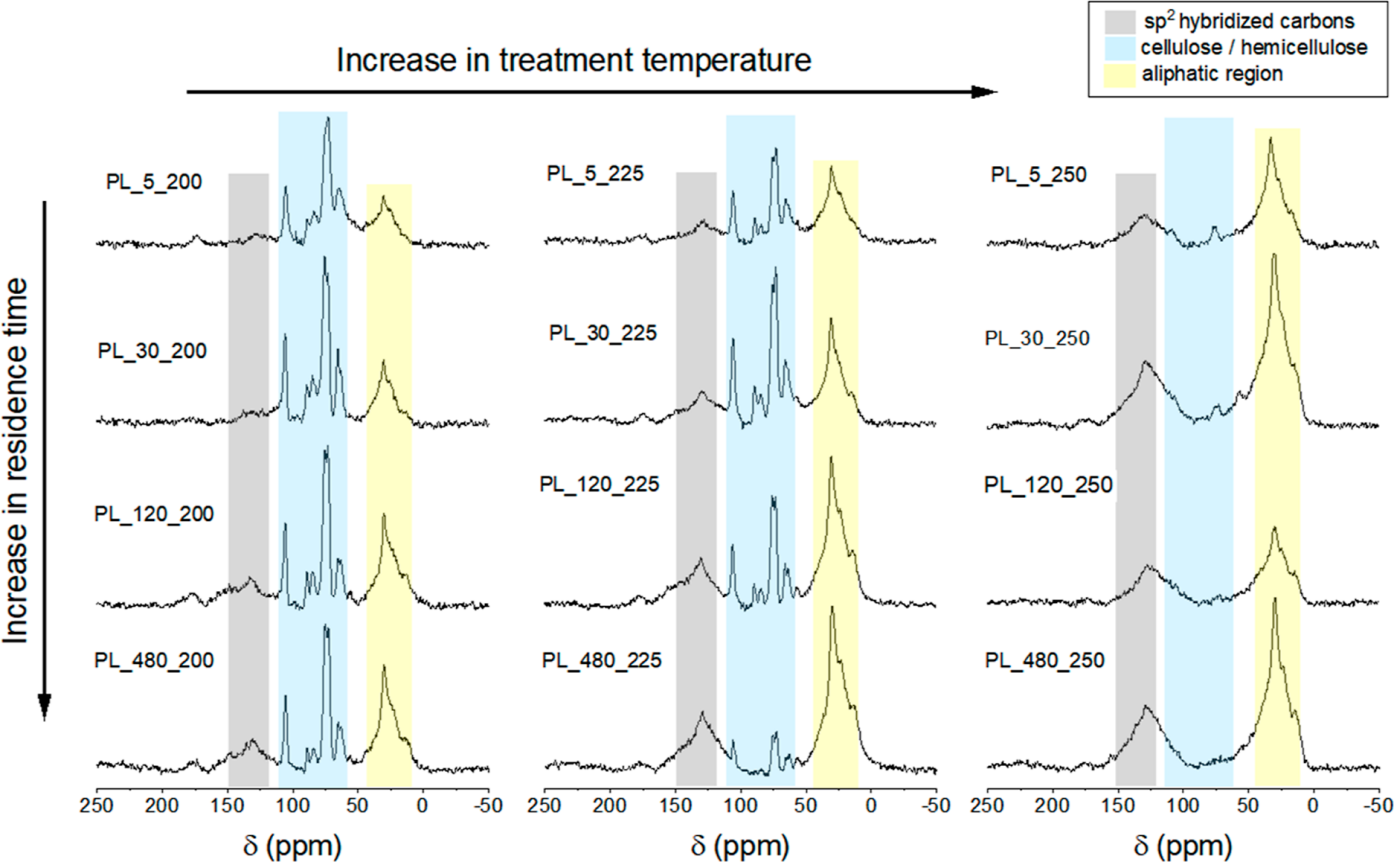
^13^C CP/MAS NMR spectra of hydrochars produced with increasing treatment temperatures (from left to right: 200, 225 and 250 °C) and increasing residence times (from top to bottom: 5, 30, 120, 480 min).

The effect of treatment temperature on hydrochars composition is illustrated by the ^13^C and ^1^H NMR spectra of samples synthesized with a residence time of 120 min (Figure 2). The residence time of 120 min was selected because Ghanim et al.^31^ concluded that hydrochars synthesized at 250 °C and 120 min exhibited higher yields and total C contents. The ^13^C NMR spectra of samples synthesized using different residence times in the range of temperatures where the HTC process caused significant chemical/structural changes (from 200 to 250 °C) are shown in Figure 3.

In the ^1^H NMR spectra of PL and the derived hydrochars (Figure 2b), it is possible to identify strong signals with chemical shifts typical of organic functional groups. Alkyl protons in fatty acid chains resonate around 1–2 ppm and present narrower signals due to their higher mobility. O-Alkyl and aromatic protons resonate around 3–5 and 6 ppm, respectively. The chemical shifts of methoxyl groups (2.7–3.0 ppm) could not be distinguished from the ones associated with carbohydrates (3.2–3.6 ppm).^38^ Notably, as the temperature increased, the peak around 4 ppm, primarily associated with O-alkyl groups in carbohydrates, decreased gradually, being completely absent for temperatures ≥250 °C; these results are in good agreement with the ^13^C NMR results, as detailed below. Similarly, the signal around 1 ppm (mainly due to alkyl protons) became somewhat less intense and broader with the increase in the hydrothermal treatment temperature. The broadened shoulder around 6 ppm corresponds to increasing amounts of aromatic protons along the carbonization process.

The ^13^C NMR spectra (Figure 2a) revealed the chemical changes occurring during the HTC of the water-suspended biomass, which led to the gradual transformation of the assigned components into aromatic and aliphatic structures.^19^ The spectrum of samples synthesized under 150 °C showed characteristics similar to the original feedstock. Structural changes begin at 175 °C, with observable hemicellulose degradation through dehydration and decarboxylation, as confirmed in the Van Krevelen diagram reported by Ghanim et al.^31^ The typical signals of hemicellulose (around 20 and 173 ppm) were the first ones to disappear upon the increase in the hydrothermal treatment temperature, which is consistent with the lower activation energy for the thermal degradation of hemicellulose in comparison with cellulose.^35,37,38^ In fact, the characteristic cellulose peaks are still present and well resolved in the ^13^C NMR spectra up to 225 °C, which aligns with studies indicating that the transformation of cellulose in hydrothermal treatments typically occurs at temperatures ≥220 °C.^35,36^ The growth of the prominent peak in the alkyl region around 30 ppm suggests the presence of aliphatic carbon chains, while the other alkyl peaks are associated with short side chains attached to, or bridges between, aromatic structures.^37,39^ The presence of these aliphatic chains has been previously identified in solid-state ^13^C NMR spectra of several carbon materials produced by low-temperature pyrolysis (below 350 °C) and HTC.^26,35,37^ Aromatic network structures with a central aromatic peak around 130 ppm started to grow at 200 °C, and the corresponding resonance became increasingly intense with the increase in HTC temperature.

The loss of lignin methoxyl groups (signal at 56 ppm in the ^13^C NMR spectra) occurred in the 225–250 °C range. In addition, the samples PL_120_275 and PL_120_300 exhibited an almost complete absence of signals corresponding to oxygen attached to aromatic C, O-alkyl C, carboxyl or methoxyl, indicating the degradation of carbohydrates was complete. The Van Krevelen diagram reported by Ghanim et al.^31^ shows these high-temperature hydrochars with O/C atomic ratios between 0 and 0.1, which aligns with the mentioned absence of oxygenated functional groups. Methoxyl is a characteristic functional group of lignin, and lignin moieties are reported to be linked through ether bonds, which suffer cleavage during the thermal process at temperatures lower than those corresponding to the loss of methoxyl groups.^39,40^ Despite the occurrence of these cleavages, the aromatic clusters of lignin are likely to remain in hydrochars during the carbonization process.^40^ The above NMR results indicate that the structural units of hydrochars synthesized above 250 °C are composed of sp^2^ hybridized carbons connected by aliphatic carbon chains, consistent with the observation of the dominant resonances around 130 and 30 ppm.^41^

PL is a heterogeneous material containing high amounts of hemicellulose sugars (close to 20% w/w), cellulose (17% w/w), and also peptides and fatty acids, as reported by Ghanim et al.^31^ It is possible to observe in Figure 2a that the ^13^C NMR spectra of the samples prepared at temperatures of 250 °C and above do not exhibit any cellulose or hemicellulose peaks, indicating the degradation of these carbohydrates during the HTC. However, given the higher stability of cellulose compared to hemicellulose, it is likely that the HTC was primarily governed by the reactivity of cellulose.^35^ One mechanism proposed for the HTC of cellulose was given by Falco et al.,^35^ who observed that during the hydrothermal treatment, the fibrous network of cellulose is disrupted into nano/microsized cellulose, forming spherical enveloped fragments with a small interface in contact with the surrounding water. In this way, cellulose undergoes a minimal degree of hydrolysis, favoring intramolecular rearrangement due to creating a homogeneous thermal environment resembling the pyrolysis process. Therefore, reactions characteristic of pyrolysis occur, including intramolecular condensation, dehydration and decarboxylation, resulting in a structure composed of condensed aromatic rings.^42^ This mechanism appears to align with the modifications observed during the thermal conversion process of PL into HCs.

Jiang et al.^19^ also reported ^1^H and ^13^C solid-state NMR spectra of PL and biochars obtained from pyrolysis of PL at increasing temperatures (from 100 to 600 °C). Biochars synthesized at 300 °C presented ^1^H and ^13^C spectra with remarkable similarity to those reported in this work for hydrochars obtained at temperatures ≥250 °C, with only a slightly higher amount of O–C_aro_ in the case of the biochars. When the pyrolysis temperature was further increased, the ^13^C NMR spectra were dominated by the aromatic signal around 130 ppm, and ^1^H NMR spectra presented gradually decreasing amounts of alkyl protons and increasing amounts of aromatic protons. Similar results were observed in the ^13^C NMR spectra of poultry manure reported by Cimò et al.,^26^ who investigated the effect of temperature and residence time of poultry manure pyrolysis on biochar properties. Biochars produced at 350 and 450 °C showed an abundance of alkyl groups and a central aromatic peak around 130 ppm in the ^13^C NMR spectra, while at 600 °C mainly aromatic carbon was present, which was formed from the simultaneous loss of aliphatic chains, carbohydrates and N-containing organic compounds. They also concluded that the chemical composition of biochars was primarily dependent on the treatment temperature rather than the residence time, which agrees with the findings obtained for the hydrochars in the present work.

While both studies indicate the significant role of the treatment temperature in determining the chemical composition of biochar, it is essential to notice the critical differences in the chemical and structural features of biochar and hydrochar samples prepared at similar conditions. For example, in the case of the PL-derived hydrochars described in the present manuscript, high amounts of cellulose are still observed after hydrothermal treatment at 200 °C for 8 h; on the other hand, much weaker cellulose signals are detected in the ^13^C NMR spectra reported by Jiang et al.^19^ for a PL-derived biochar prepared at the same temperature and with the same residence time.

The formation of methylene chains and condensation into aromatic rings during HTC occurs intensely from 200 to 250 °C, with a reduction in the proportion of cellulose in the total carbon signal and a relative increase in the proportion of aromatic and aliphatic carbons. At 200 °C, the reaction kinetics of HTC is much slower than at 225 °C, where the residence time had a stronger influence on the dehydration and aromatization process of hydrochars (Figure 3). At 225 °C, up to 120 min, peaks related to carbohydrates (from 50 to 110 ppm) were significant, showing the presence of thermally untransformed or incompletely transformed biomass; when the treatment time reached 480 min, the aromatic resonance became more substantial than the carbohydrate peaks. As for treatment temperatures ≥250 °C, all the spectra were dominated by the alkyl and aromatic regions, although the spectra of samples with residence times of 5 and 30 min still showed weak signals related to cellulose. Given that lignin is minimally affected by hydrothermal treatment and its primary function is to support the plant cell wall, it has been shown that lignin can stabilize cellulose and prevent its disruption during HTC; this stabilization shifts the degradation of cellulose to higher temperatures or longer residence times.^10,43^ Based on these observations and considering the energy efficiency of synthesizing PL hydrochars, the optimal conditions for HTC are temperatures between 225 and 250 °C and residence times exceeding 120 min. It is worth noting that this finding agrees with a previous study on HTC of wheat straw digestate.^44^

The results discussed above also agree well with previous work on HTC of PL under different temperatures (180, 200, 220 and 250 °C) and residence times (5, 30 and 60 min). The authors reported obtaining hydrochars with low oxygen content and significant clustering observed only for samples treated at 250 °C. They also observed that a portion of the nitrogen content from PL remained in the solid phase.^45^ The remaining nitrogen compounds might be contributing to the signal around 175 ppm (N–C=O) in the ^13^C NMR spectra reported in the present work.

Reza et al.,^44^ on the effects of HTC temperature and residence time on hydrochars of wheat straw digestate, reported that the precursor showed a typical ^13^C NMR spectrum of a natural lignocellulosic material. Insofar as the temperature was raised from 180 to 220 °C, a reduction of cellulose in the total carbon signal was observed, along with a relative increase of aromatic and aliphatic carbon. Like in HTC of PL, the aliphatic peak around 30 ppm presented stability throughout the HTC process. Nevertheless, even in hydrochars synthesized at 260 °C for 6 h, they noted the presence of distinct and strong peaks at chemical shifts of 56 ppm from methoxyl, 147 and 152 ppm from aromatic C bonded to oxygen, and at 133 ppm from aromatic C bonded to propyl chains in guaiacyl and syringyl units of lignin. The effects of the HTC process on wheat straw, olive residues and poplar wood biomasses at 180, 210 and 230 °C (480 min as residence time), studied by Wiedner et al.,^46^ also showed ^13^C NMR spectra with prominent broad peaks around 130 and 30 ppm, along with sharp peaks at chemical shifts related to O-aryl and methoxyl groups. The authors reported decreasing amounts of lignin during the HTC process, although yet present in hydrochars synthesized at 230 °C. The mentioned results differ from the spectra presented in this work, as those precursors contain significantly higher amounts of lignin in comparison with the PL precursor analyzed here. As mentioned, PL-derived hydrochars presented a less heterogeneous matrix, with no lignin-related signals detected when the treatment temperature was ≥250 °C. The broad aromatic peak observed could be assigned to a mixture of aromatic moieties, encompassing protonated, condensed or alkyl-bearing rings.

**Figure 4:**
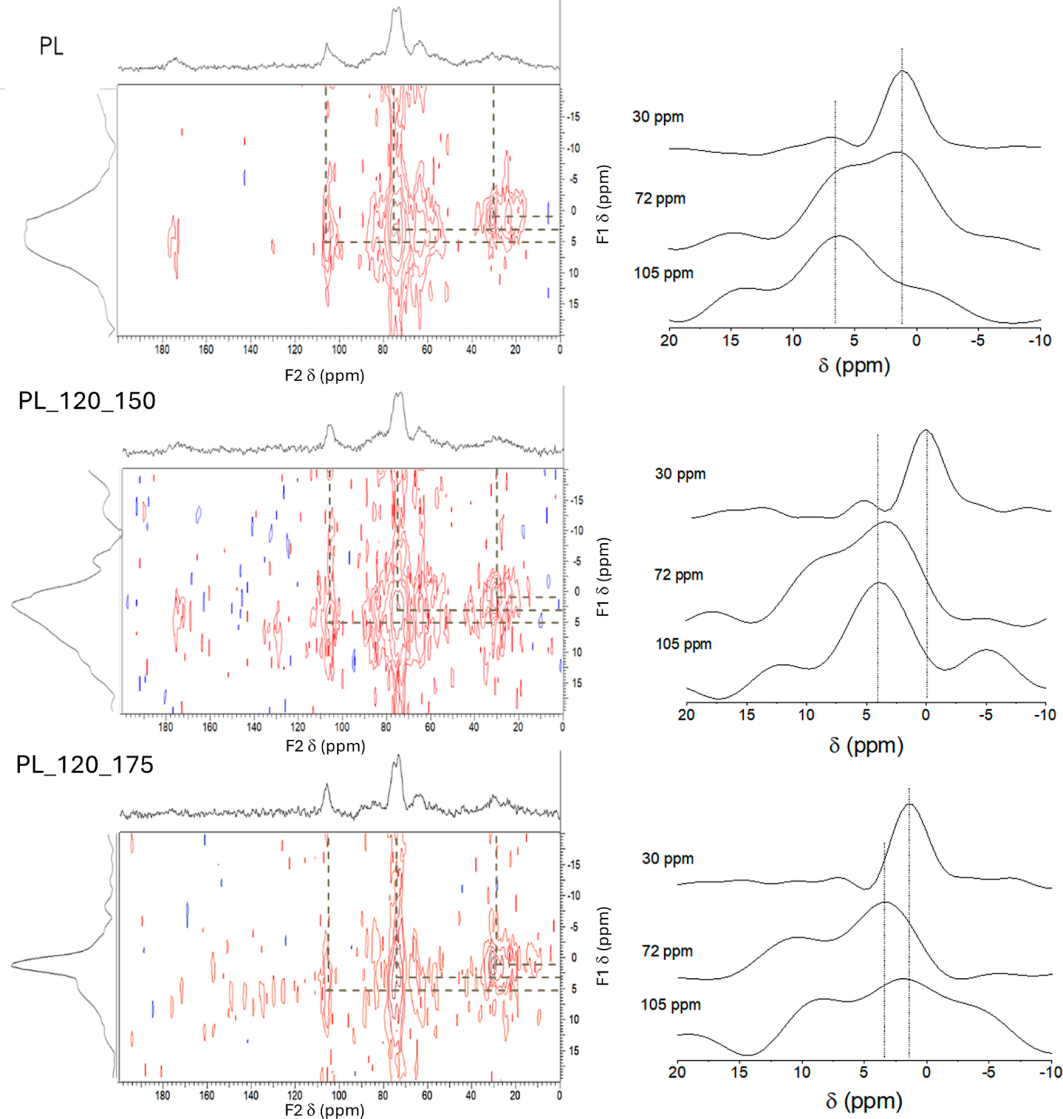
^1^H–^13^C 2D WISE spectra of raw PL and hydrochars produced at 150 and 175 °C (from up to down) and 120 min of residence time. The ^1^H wide-line slices corresponding to different ^13^C chemical shifts are shown on the right of each 2D spectrum.

Figure 4 shows the 2D WISE spectra of PL and some representative hydrochars and their projections along the ^13^C (F2, horizontal) and ^1^H (F1, vertical) dimensions. In order to investigate possible connectivities of different functional groups, some ^1^H wide-line slices were selected along three ^13^C chemical shifts: 105 and 72 ppm, associated with O-alkyl signals due to carbohydrates, and 30 ppm, associated with CH_2_ groups in methylene chains. The lineshapes of the three projections are not identical; the mobile side chains of methyl groups in the organic matter, along with the CH_2_ and CH_3_ groups in fatty acid chains, gave rise to narrower lines in the ^1^H dimension correlated to the 30 ppm ^13^C resonance, as a result of the reduced average effect of the homonuclear dipole coupling associated with the motion of these groups. The other components along the ^1^H dimension were broader, with nearly Gaussian line shape, associated with large homonuclear dipole couplings in the rigid structures of O-alkyl groups. The 2D WISE experiments, therefore, reveal that the ^1^H linewidths associated with the individual ^13^C resonances are not identical and indicate that there are domains in the hydrochars with different mobility. The ^1^H lineshapes of the methylene chains confirm the higher mobility of these chains when compared to O-alkyl or aromatic groups.

It is interesting to compare the ^1^H slices shown in Figure 4 with the corresponding 1D ^1^H NMR spectra obtained for the same samples, shown in Figure 2. When a resonance is observed in the direct 1D measurement but not in any projection of the 2D spectra, it suggests that the H-containing group accountable for that resonance is ineffective in transferring the polarization to nearby ^13^C nuclei. This inefficiency can stem from either a lack of ^1^H–^13^C dipole coupling (owing to substantial internuclear distances) or molecular motion (resulting in diminished average dipole coupling).^47^ In the case of the spectra shown in Figures 2 and 4, it is clear that the narrowest lines close to 1–2 ppm in the 1D ^1^H NMR spectra do not appear either on the slices or the overall projections of the 2D WISE spectra; the ^1^H slices corresponding to a ^13^C chemical shift of 30 ppm do exhibit some resonances around 1–2 ppm, but with a much larger line width in comparison with the 1D spectra. This observation indicates that the mobile groups responsible for the narrow ^1^H resonances in the 1D NMR spectra (primarily associated with mobile fatty acids chains in PL) are ineffective in transferring polarization to the ^13^C nuclei nearby. The low efficiency of ^1^H–^13^C polarization transfer during the CP/MAS experiments is commonly observed in substances exhibiting high molecular mobility, as is the case of fatty acid chains.^48,49^ This is due to the averaging of the ^1^H–^13^C heteronuclear dipolar coupling associated with molecular motion.

Cao et al.^37^ reported 2D ^1^H–^13^C HETCOR NMR analysis of hydrochars obtained from swine manure; the obtained spectra showed that alkyl, O-alkyl and aromatic carbons were primarily correlated with their directly attached protons, and chemical shifts of the ^1^H resonances were similar to those obtained in the present work - the ^1^H signal around 1.5 ppm derived from protons of alkyl carbons at 29 ppm, protons resonating at approximately 4 ppm were associated with O-alkyl carbons, while aromatic carbons were correlated with protons at 7 ppm.

The intensities of the signals associated with aromatic and carboxylic carbons in the spectra obtained for PL and the low-temperature hydrochars were relatively low; therefore, the corresponding ^1^H slices presented very low signal/noise ratios and were not shown in Figure 4. In addition, the ^13^C signals at the chemical shifts of 72 and 105 ppm showed correlations with the aromatic ^1^H signals (6–7 ppm), which indicates an effective polarization transfer from aromatic protons to ^13^C nuclei in carbohydrates, as was shown by Le Brech et al.^38^ Considering that the WISE experiment investigates correlations between nearby ^13^C and ^1^H nuclei, facilitated by heteronuclear dipole coupling, the findings affirm the intimate association of carbohydrates with lignin in PL and low-temperature hydrochars. It is also worth noting that spin diffusion acts to spread polarization within the system of dipolar coupled ^1^H nuclei in rigid spin networks, as is the case of the structure of carbohydrates, lignin and the hydrochars; thus, even ^13^C nuclei not directly coupled to a given H-containing chemical group can show some correlation with this group in 2D WISE spectra, depending on the contact time used in these experiments.^29^ Another study utilizing the 2D WISE technique to analyze dry humic acids demonstrated that the linewidths of ^1^H resonances from O-alkyl carbohydrates were broader than those from aromatic protons. Since ^1^H–^1^H dipolar couplings are reduced due to segmental mobility, the large linewidths in signals associated with carbohydrates suggest there were no fast, large-amplitude motions in these domains.^50^

### 2.2 Analysis of P-Containing Groups

#### 2.2.1 ^31^P SPE/MAS NMR: Effects of Temperature and Residence Time on P-Containing Groups of Hydrochars.

**Figure 5:**
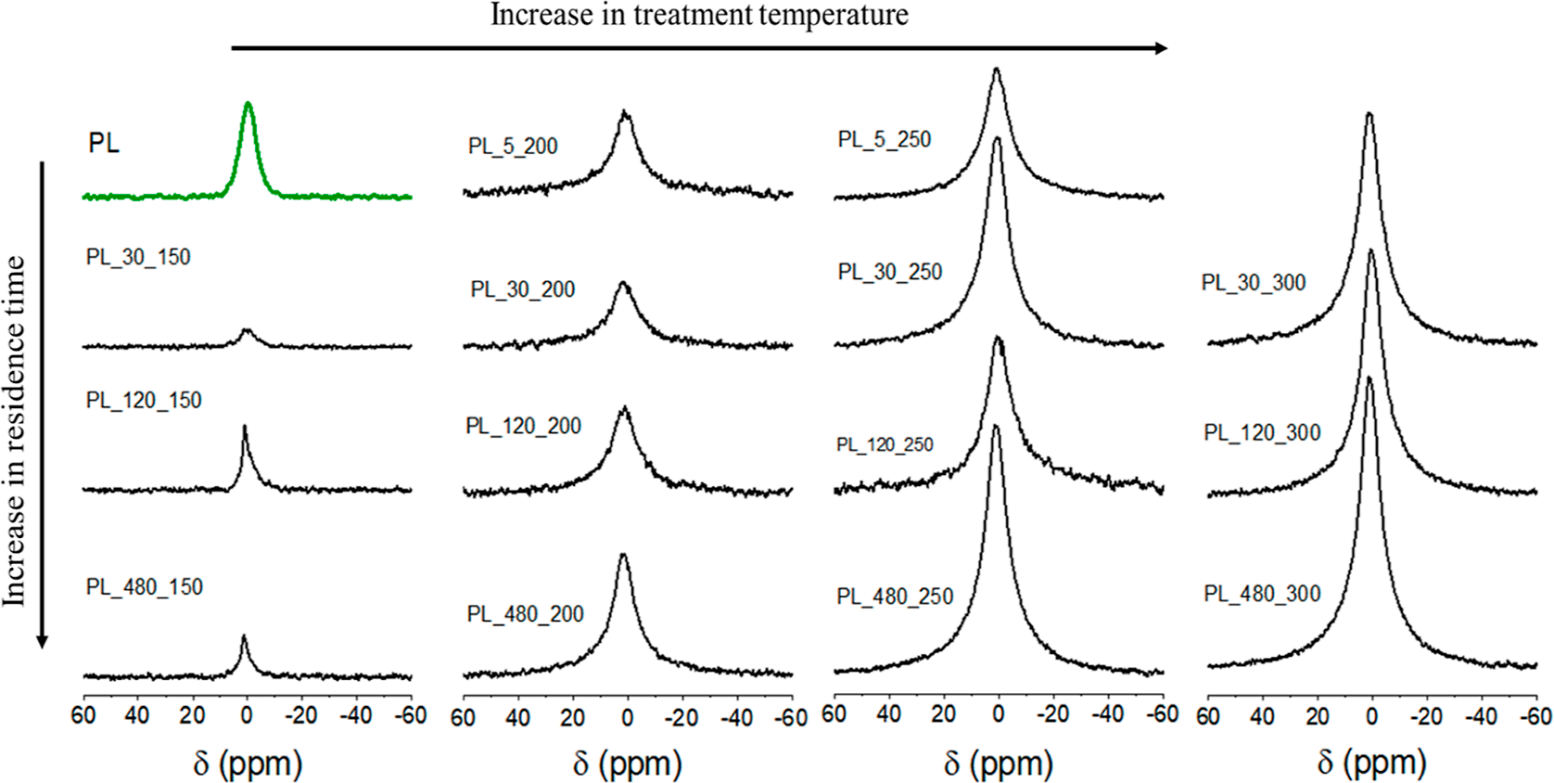
^31^P SPE/MAS NMR spectra of PL (in green) and hydrochars produced at increasing treatment temperatures (from left to right: 150, 200, 250 and 300 °C) and increasing residence times (from top to bottom: 5, 30, 120 and 480 min).

The ^31^P SPE/MAS NMR spectra reflect the various P species of each sample quantitatively; the spectra of PL and PL-derived hydrochars obtained by HTC at increasing temperatures and residence times are shown in Figure 5. Most spectra exhibited a broad signal around 0 ppm, regardless of the temperature and residence time. The samples synthesized at 150 °C were an exception, displaying a relatively narrower peak at 1 ppm. All samples treated for 480 min exhibited increased ^31^P SPE/MAS NMR spectral intensities compared to those treated for shorter residence times, irrespective of the temperature. Additionally, higher temperatures resulted in further increases in peak intensities. The increase in peak intensities in the ^31^P NMR spectra indicates higher phosphorus content in the hydrochars, which is attributed to greater phosphorus uptake and immobilization during the HTC process.

The chemical shift around 0 ppm can be primarily assigned to orthophosphates, with narrow peaks featuring a Lorentzian line shape indicating better-ordered structures. In contrast, the Gaussian line shape typically suggests a range of similar chemical environments that cannot be separated into clearly distinguishable chemical shifts. Therefore, this broad peak encompasses all P species, including inorganic phosphate complexed with various metal cations and the main organic phosphates found in PL, such as undigested phytic acid and compounds derived from metabolic processes.^51^

Han et al.^18^ demonstrated that hydrochars derived from sewage sludge, synthesized at temperatures ranging from 140 to 200 °C and residence times of 10–60 min, exhibited solid-state ^31^P NMR spectra featuring a single broad signal spanning from 15 to −15 ppm. Notably, no discernible differences were observed between samples from various treatment temperatures, and the P species were assigned primarily as orthophosphates interacting with Fe, Ca, and Al.

The recorded ^31^P NMR spectra of samples subjected to HTC at different pH, where two different kinds of acids were tested, are shown in Figure S1 (Supporting Information). These spectra showed the same general features as the other samples without initial pH modification, presenting broad signals around 0 ppm. Ghanim et al.^30^ reported that up to 40% of the ash content was removed through acidification with sulfuric acid (SA), which explains the significant reduction in the ^31^P NMR signal intensity for the sample treated with SA at an initial pH of 2. The acidic pH likely enhanced the solubilization of phosphates from PL and hindered Ca and Mg phosphate precipitation, typically favored by higher pH levels.

While the SPE NMR spectra record the direct polarization of the nuclei in question, the ^1^H–^31^P CP draws magnetization from neighboring ^1^H species, emphasizing ^31^P sites close to protons. This polarization transfer from abundant and high-sensitivity nuclei (^1^H) to less abundant nuclei (^31^P, in this case) relies on a robust static component of the dipolar coupling between the proton and ^31^P nuclei. Therefore, CP/MAS NMR spectra provide information about P-containing groups closely associated with hydrogen.^52,53^ Comparing SPE and CP spectra can thus offer a deeper understanding of the types of phosphates formed during the HTC process.

#### 2.2.2 ^31^P CP/MAS NMR: Effects of Temperature and Residence Time on Protonated Phosphates of Hydrochars

**Figure 6:**
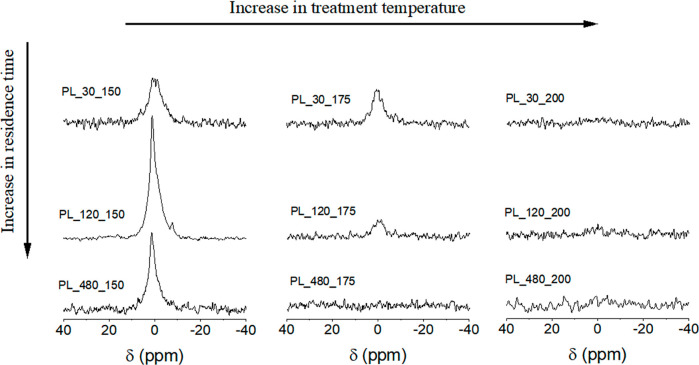
^31^P CP/MAS NMR spectra of hydrochars produced at increasing treatment temperatures (from left to right: 150, 175 and 200 °C) and increasing residence times (from top to bottom: 30, 120 and 480 min).

The ^31^P CP/MAS NMR spectra of hydrochars synthesized up to 200 °C and residence times of 30, 120 and 480 min are presented in Figure 6. It is observed that higher treatment temperatures result in decreased signal intensity detected through the CP sequence. Notably, no ^31^P NMR signal is observed for samples treated at temperatures ≥200 °C, in contrast to the results obtained from the SPE sequence. Furthermore, the residence time had a more pronounced effect on samples treated at 175 °C, as evidenced by a significant decrease in peak intensities with increasing residence time at this temperature. This suggests that 175 °C is the temperature at which P species closely associated with hydrogen are highly solubilized.

Huang and Tang^54^ reported similar findings when comparing solid-state CP and SPE ^31^P NMR spectra of hydrochars produced from HTC of activated sludge at 225 °C for 24 h. The SPE spectrum of the hydrochar showed a single predominant peak centered at −15 ppm, primarily attributed to orthophosphate. On the other hand, in the CP spectrum of the activated sludge hydrochar, no P signal was observed, despite the precursor containing protonated phosphate, which should have been detectable using the CP sequence. The low P observation in the hydrochar by solid-state NMR led the authors to conclude that all P species were hydrolyzed into orthophosphate during this thermal treatment, forming different phosphate salts or associated with other minerals.

The P speciation results presented by Ghanim et al.^32^ showed that Ca and K are the major nutrients present in PL, with Ca/P and K/P atomic ratios >1, followed by Mg and Na in minor quantities. Other elements, such as Mn, Al, Cu, Zn, and Fe, constituted a minor fraction, thus playing a less significant role in phosphorus mobility. Additionally, Fe and Mn phosphates are unlikely to be detected by NMR due to paramagnetic line-broadening. In hydrochars synthesized at low temperatures, significant amounts of K were present and correlated with water-soluble phosphorus (WSP). Conversely, the concentrations of Ca and Mg in the hydrochars increased with higher treatment temperatures, particularly above 200 °C for residence times of 30 and 120 min, and above 175 °C for residence time of 480 °C. The authors also observed that, starting from 200 °C, P recovery by the hydrochars increased from 60 to 100%, while WSP decreased from over 50% to approximately 2%.

The increased amount of metals in high-temperature hydrochars is primarily due to the reduced yield of hydrochar as the HTC temperature rises. During the hydrothermal process, a portion of the organic fraction of the biomass is lost, resulting in the remaining inorganic fraction constituting a larger mass percentage in the hydrochars. Further quantitative details of metals, organic fraction and ash content can be found in the work of Ghanim et al.^30−32^ Another reason for the increase of the metal contents and phosphorus in high-temperature hydrochars is related to the precipitation of inorganic species that may have been solubilized at the beginning of the hydrothermal process, as occurred with P in hydrochar samples prepared below 200 °C. These mentioned findings suggest that stable forms of phosphates precipitated at temperatures above 200 °C. The stable phosphates formed may consist of Ca- and Mg-containing compounds, such as anhydrous dicalcium phosphate (CaHPO_4_) and amorphous Mg phosphates, which are known to resonate around 0 ppm. These compounds are likely the forms of phosphate present in high-temperature hydrochar samples.^51,55−57^

As is common in many heterogeneous materials, broad NMR peaks usually indicate the presence of amorphous or poorly ordered phases. For the distribution of different local chemical environments, peak broadening can also be caused by structurally distorted unit cells of the same compound at a surface, or by chemisorbed forms that do not resemble any known crystalline or bulk amorphous material.^58^ The XRD results of representative hydrochar samples confirmed the amorphous character of the hydrochars, as shown in Figure S2 (Supporting Information). Therefore, given the somewhat broad nature of the ^31^P NMR lines, P species PL-derived hydrochars are unlikely to exist in crystalline phases, which is consistent with previous findings that indicated hydrothermal treatment to generally homogenize all P forms into orthophosphates, forming disordered and amorphous phases.^18,54^ HTC of swine manure, for example, was reported to positively affect the crystallization of phosphates at low temperatures and short residence times. However, as the reaction severity increased, crystallization was hindered, probably due to the formation of amorphous carbon, which favored the adsorption of P by the porous hydrochar.^11^

Chemical shifts of phosphate groups are influenced by the number of cations and protons bonded to phosphate, cation electronegativity and the presence of hydrating water.^23^ Protonation of phosphates is reported to result in an upfield peak shift and an increase in the chemical shift anisotropy.^25^ Among the metal cations present in PL, Ca is a crucial element for capturing P in hydrochar due to its high concentration and strong affinity for P, leading to the formation of precipitates and surface complexes.^11,54^ Calcium orthophosphates can exist in many different forms and, consequently, exhibit different ^31^P chemical shifts; for example, HPO_4_^2–^ of dicalcium phosphate dihydrate resonates at 1.4 ppm,^59^ while octacalcium phosphates, amorphous calcium phosphates, and tribasic calcium phosphate resonate around 3 ppm.^51,53,58^

In the ^31^P SPE/MAS NMR spectra of PL hydrochars, although the peak around 0 ppm can generally be assigned to orthophosphates, its broad line shape suggests that it might contain contributions due to different types of Ca orthophosphates, once hydroxyapatite, octacalcium phosphate, and tribasic calcium phosphates are species reported to be found in hydrochars derived from materials rich in Ca and P.^11,18^ This possibility is reinforced by observing the spectra of samples synthesized with initial pH of 4 and 2 (Figure S1), which show a slight shift toward lower frequencies, probably caused by the hampered precipitation of Ca phosphates at lower pH.^60^ As the second major nutrient encountered in the PL-derived hydrochars, Mg seems to have had an essential role in the phosphorus speciation of PL during HTC. Magnesium can inhibit precipitation and crystallinity of Ca phosphates, allowing the formation of amorphous calcium phosphate due to Mg^2+^ incorporation into the Ca phosphate structure.^61^

The absence of signals in the CP/MAS spectra for temperatures above 200 °C indicates the sorption or precipitation of unprotonated phosphates - anhydrous compounds like Ca_3_(PO_4_)_2_ and Mg_3_(PO_4_)_2_, which contain no structural hydrogen. Amorphous Mg_3_(PO_4_)_2_ was reported to resonate around 0.5 ppm, whereas the crystalline phase resonates around −0.5 ppm.^57,62^ Another possibility is the formation of sorbed forms of phosphates. Hinedi et al.^58^ demonstrated that surface-sorbed phosphate groups on Ca carbonate exhibited a ^31^P chemical shift around 3 ppm and were not directly protonated, resulting in a low signal-to-noise ratio in the CP spectrum. Detection was possible only because a small fraction of the phosphate groups had weak dipolar couplings to nearby protons. These species gave rise to ^31^P NMR peaks with larger line width than well-crystallized compounds, but narrower than the values reported for the amorphous Ca phosphates.

#### 2.2.3 ^31^P NMR Spectra of Water-Soluble Phosphates

**Figure 7:**
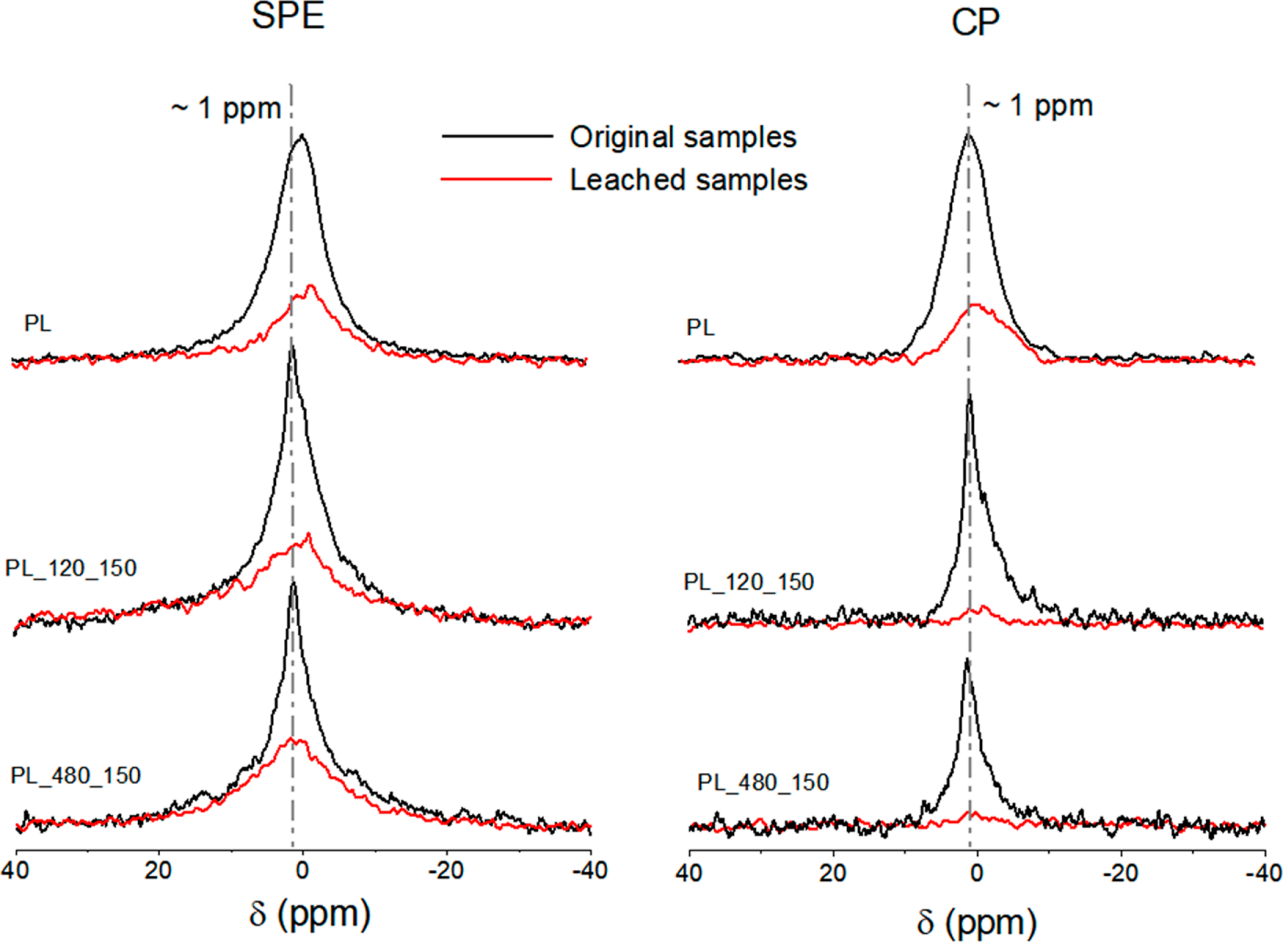
^31^P SPE/MAS (left) and CP/MAS (on the right) spectra of raw PL (top) and hydrochars produced at 150 °C, 120 min (middle) and 480 min (bottom) of residence time. Black lines indicate the spectra obtained for the original samples; red lines correspond to the spectra recorded after the water extraction procedure.

The ^31^P CP/MAS NMR spectra and the phosphorus speciation results presented by Ghanim et al.^32^ indicate that the detected signals are predominantly associated with water-soluble species, mainly composed of K-containing phosphates. These findings suggest that the K phosphates are protonated, whereas the other species likely consist of orthophosphates primarily bonded to cations with minimal hydrogen content. A water-extraction procedure was carried out to confirm this assumption, and the solid residues from the extraction—identified as leached samples—were analyzed again by ^31^P NMR using both sequences, CP and SPE. These spectra are shown in Figure 7.

The ^31^P SPE/MAS NMR spectra of the leached samples showed peaks considerably weaker and somewhat broader than those obtained for the original samples. This observation agrees with the results reported by Ghanim et al.,^32^ which showed that around 50% of the total P content of these samples was water-soluble. Noticeably, after water extraction, the ^31^P NMR spectra of PL and PL_150_120 samples presented a maximum at −1 ppm, slightly shifted to lower frequencies. For PL_150_480 leached sample, the signal remained centered at 1 ppm and was broader in comparison with the ones corresponding to PL and PL_150_120, indicating that other insoluble phosphate species were precipitated during the HTC at 150 °C for 480 min.

The ^31^P CP/MAS NMR spectra of the leached hydrochar samples showed that ^31^P nuclei in close interaction with ^1^H nuclei were largely removed after water extraction; this suggests that the CP signals were mainly due to weakly sorbed phosphate species. Conversely, in the ^31^P NMR spectrum of PL, certain P species from the precursor remained after water extraction, particularly those contributing to the lower frequency signals of the broad peak around 0 ppm. This suggests the PL sample contains P species closely associated with hydrogens and not water-soluble, with resonances between 0 and −7 ppm, which can be assigned to pyrophosphates and organic phosphorus.^17,63^ Furthermore, it is interesting to note that most of the P species in PL correspond to ^31^P nuclei closely associated with ^1^H nuclei, given the substantial similarity observed between the CP and SPE spectra for the PL sample (both before and after the water extraction procedure).

The CP spectrum of the leached PL sample likely included a significant contribution of organic P. In a study investigating different P contents of soil samples using solid-state ^31^P NMR, Dougherty et al.^64^ demonstrated that ^31^P CP/MAS NMR could not detect inorganic P. Instead, organic P species produced broadened resonances around −1.6 ppm, accompanied by prominent sidebands. At the same time, the ^31^P SPE/MAS NMR spectra presented a sharp peak at 2.7 ppm, associated with inorganic P, besides a small shoulder upfield, associated with the organic P fraction. Phytate is the main form of organic P found in animal manure, PL and soils; it consists of a six-C ring with 1 H and 1 phosphate attached to each C, and these phosphates usually interact with various metal ions from the environment to form soluble and insoluble phytate salts.^65^ The possible metal phytates present in the PL sample of the present work are K phytate and Ca phytate, both of which are related to a broad peak resonating around 0 ppm in the spectra.^63^ Once these metal phytates are soluble in an acidic environment,^66^ the organic phosphorus quantified using the SMT was possibly underestimated by Ghanim et al.^32^ (∼0.9 mg/g) once these species are likely to represent a considerable fraction of P present in the PL sample.

McDowell et al.^67^ used ^31^P CP/MAS NMR to show that water-extracted species of soil samples could be assigned to loosely sorbed protonated Ca phosphates. The spectra had a broad signal around 0 ppm before extraction, composed mainly of Ca (downfield) and Al phosphates (upfield); only the resonance due to Al phosphates remained after extraction. Similar results were found by Lookman et al.^68^ with a fast desorbing P pool giving a sharp peak in the CP spectrum at 1.16 ppm, which was absent in the spectrum of the same soil sample after leaching. This peak was assigned to a readily soluble Ca phosphate phase (not condensed) or ‘loosely’ adsorbed (protonated) P. A signal at 1.5 ppm was also observed with both SPE and CP sequences in a sewage sludge sample, absent after the water extraction, and was attributed to brushite (CaHP0_4_·2H_2_0).^55^

Despite the complexity of the material, samples synthesized at 150 °C presented ^31^P NMR spectra with narrower line width when compared to hydrochars produced at higher temperatures. Besides, the disappearance of the narrow peak upon water extraction (see Figure 7) indicates the occurrence of inorganic phosphate bonded to physically adsorbed water through hydrogen bonds, which are readily water-soluble species. Na orthophosphate (NaH_2_PO_4·_H_2_O) is reported to give a ^31^P NMR peak at 1 ppm.^23,69^ K phosphate in the form of KH_2_PO_4_ resonates at around 4 ppm,^63^ and it was not possible to assign the sharp peak close to 1 ppm to a specific inorganic K phosphate. However, chemical shifts of heterogeneous and amorphous compounds might differ from those of crystalline phases since the redistribution of electronic density around the ^31^P nucleus is known to change the values of the corresponding chemical shifts.^53,56,57^

In contrast with the ^31^P NMR spectrum obtained from the PL sample in the present work, Hunger et al.^51^ presented ^31^P SPE/MAS NMR spectra of PL with two sharp peaks, one around 6.5 ppm, assigned to struvite, and another around 3 ppm, assigned to phosphate sorbed to the calcite surface, besides a broad Gaussian peak centered between −10 and 1 ppm. In the CP spectra of the same samples, the peak corresponding to struvite was enhanced, and the broad signal around zero was still present, while the peak at 3 ppm was suppressed. Jiang et al.^19^ also presented ^31^P SPE/MAS NMR spectra of PL with an intense peak at 6.1 ppm and a broad shoulder containing two notable peaks centered around 2.6 and 0.6 ppm, which were assigned to Na phosphate (orthophosphate), Ca phosphate (poorly crystalline hydroxyapatite), and organic phytates (Ca or Mg phytates), respectively. In their investigation of PL carbonization during pyrolysis, researchers found that when PL underwent thermal treatment at 100 °C, the peak at 6.1 ppm disappeared. Instead, a broadened signal around 0 ppm became more prominent, and two peaks at 2.6 and 0 ppm remained. This transformation was further accentuated when employing the CP sequence. The increase in the pyrolysis temperature to 300 °C led to a spectrum with a broad peak of around 3 ppm, containing mainly apatite phosphorus, phytates and orthophosphates; when the temperature reached 600 °C, the species present were hydroxyapatite (at 2.8 ppm) and farringtonite (crystalline Mg_3_(PO_4_)_2_).

The absence of sharp peaks related to struvite (∼6 ppm) or crystalline phases in PL can be explained by the higher quantities of Ca and K (27 and 38 mg/g, respectively) present in the precursor, in addition to the performed procedures of drying and storage.^70^ On the other hand, phosphorus speciation during HTC differs from the pyrolysis process, once the aqueous medium at high temperature and pressure favors hydrolysis, solubilization and subsequent precipitation of phosphorus species. During the initial stage, the organic matter rich in carboxyl and hydroxyl groups may have coordinated with Ca and Mg, hindering the contact between these metal cations and phosphates.^71^ The subsequent degradation of the organic matter, the loss of hydroxyl groups, and the formation of stable aromatic carbon and alkyl chains occur during HTC and are expected to contribute to phosphate bonding to the available cations.

It is important to highlight that the results presented in this work can be related to various applications. Stable forms of phosphates present on the surface of hydrochars, detectable by the SPE sequence, are associated with the adsorption of heavy metals, primarily lead (Pb).^72,73^ In contrast, the CP/MAS ^31^P NMR characterization of hydrochars showed that only organic and water-soluble phosphates were detected. This finding is significant as it relates to the bioavailability of phosphorus species, with WSP indicating readily available phosphorus sources for plants.^74^

## 3 Conclusions

The solid-state ^1^H, ^13^C, and ^31^P NMR characterization of PL and its derived hydrochars provided a better understanding of the chemical and structural changes involving the HTC process of this precursor. The ^13^C NMR analysis showed that, from 250 °C, all PL hydrochars were primarily composed of alkyl and sp^2^ aromatic domains, regardless of the residence time. The primary degradation of carbohydrates occurred close to 225 °C, where the NMR spectra of hydrochars showed substantial differences as a function of the residence time. A decisive component of the HTC process is cellulose, due to its higher thermal stability compared to hemicellulose. Furthermore, the spectra suggested straightforward aromatization from the degraded carbohydrates. The 2D ^1^H–^13^C WISE spectra showed the higher mobility of alkyl domains compared to O-alkyl protons.

The SMT protocol for the speciation of phosphorus carried out by Ghanim et al.,^32^ combined with the results obtained from ^31^P SPE/MAS and CP/MAS NMR spectra, could help the understanding of phosphate transformation during HTC. The solid-state ^31^P NMR spectra showed that the water-soluble phosphates are likely to be protonated species, differing from the stable and insoluble forms of phosphates. The protonated P species detected with the CP sequence were present only in raw PL and low-temperature hydrochars, which could be assigned to organic and water-soluble phosphate forms. These labile phosphates are likely already present in PL and have not yet been hydrolyzed in the low-temperature hydrochars. As long as the temperature was raised, the hydrolysis of phosphates and precipitation or sorption of more stable forms occurred, leading to the predominance of unprotonated P species in hydrochars produced above 200 °C. All the spectra presented a broad peak around 0 ppm, assigned to orthophosphates. Although Ca was present in considerably higher amounts than any other nutrient in high-temperature samples, Mg is also present in hydrochars in significant quantities. Therefore, the broad signal around 0 ppm may overlap signals from Ca-phosphates, such as hydroxyapatite or amorphous Ca phosphate, with amorphous Mg phosphates or compounds with Mg incorporated into the Ca phosphate structure.

## 4 Materials and Methods

### 4.1 Material

**Figure 8:**
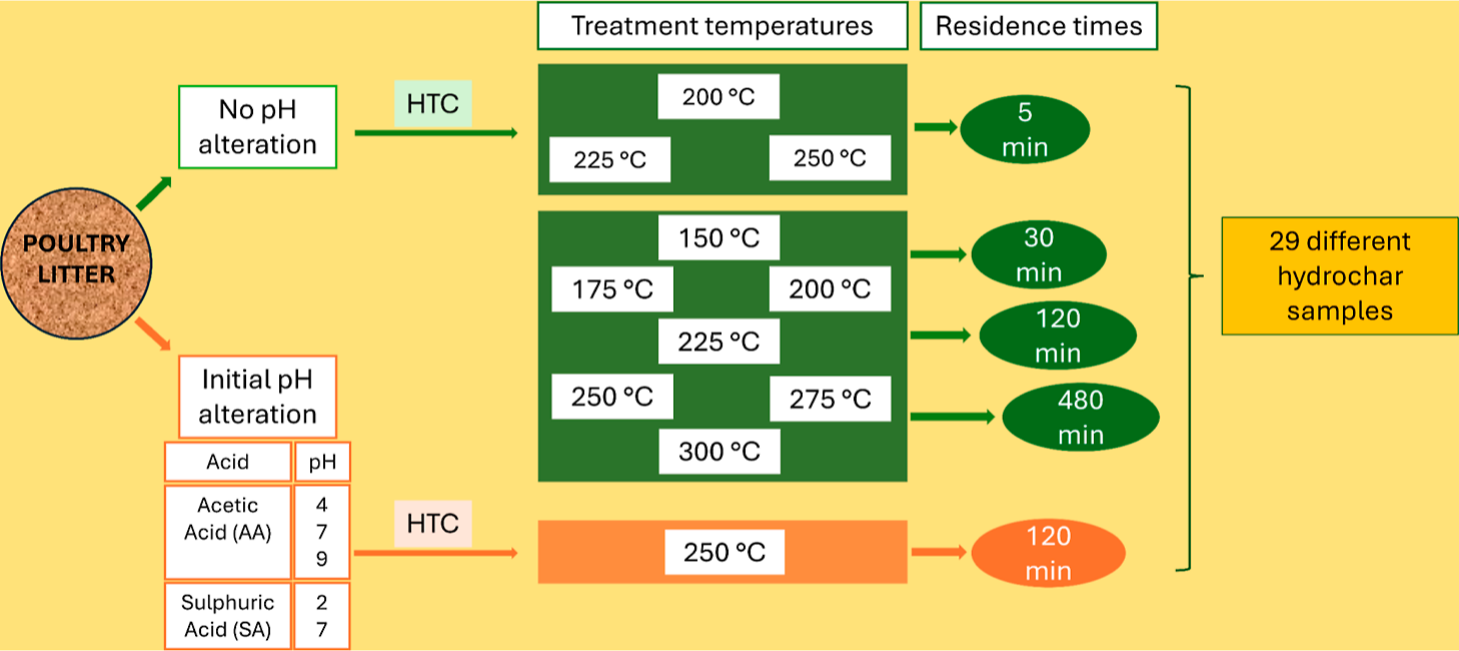
Hydrochar production schematic diagram.

The PL HCs analyzed in this work were synthesized by Ghanim et al.^30−32^ in previous works, using PL collected from a farm near Limerick, Ireland; the details of the HTC experimental procedure can be found elsewhere.^30−32^ A schematic diagram of the hydrochar production is presented in Figure 8. Briefly, at natural PL pH (8.83), the HCs were prepared using different treatment temperatures (150, 175, 200, 225, 250, 275, and 300 °C) and residence times (5, 30, 120, and 480 min). Later, the initial pH was modified by preparing HCs at a treatment temperature of 250 °C and residence time of 120 min, in the presence of acetic acid (AA) at pH of 9, 7, and 4, or the presence of SA at initial pH of 7 and 2. The samples were identified as PL_X_Y, where X is the residence time, and Y is the treatment temperature of the HTC process. Samples treated with acids were named PL_XY, where X is the acid type, and Y is the pH value of the initial solution.

### 4.2 Solid-State NMR Experiments

All the received samples were analyzed through solid-state ^1^H, ^13^C, and ^31^P NMR in order to probe the chemical environment of these nuclei in PL and the PL-derived hydrochars and to assess the chemical/structural changes promoted by the HTC processes. The NMR experiments were conducted at room temperature in a 400 MHz Varian-Agilent spectrometer operating at a magnetic field of 9.4 T at operating frequencies of 161.9, 100.6 and 399.8 MHz for ^31^P, ^13^C and ^1^H, respectively, and using a triple-resonance probe head. Powdered samples were packed into 4 mm diameter zirconia rotors for MAS experiments at 10–14 kHz spinning rate.

The SPE method was used to record ^1^H and ^31^P NMR spectra, whereas CP was used for ^13^C and ^31^P. Differences between ^31^P SPE and CP NMR spectra would provide information about the phosphorus species in question—the magnetization transfer from protons to ^31^P nuclei allows only phosphorus species close or bonded to ^1^H nuclei to be detected using the CP sequence. Two-dimensional (2D) WISE experiments were also performed to probe ^1^H–^13^C and ^1^H–^31^P heteronuclear correlations. This method allows the identification of which ^13^C and ^31^P NMR peaks (along the F2 dimension) are strongly correlated to ^1^H signals (along the F1 dimension).^29^ A sufficient number of scans was adopted to give an acceptable signal-to-noise ratio, whereas contact times and recycle delays were optimized to provide enough magnetization transfer and to avoid saturation problems, respectively. The parameters used in the SPE, CP, and 2D WISE experiments are summarized in Table 1.

**Table 1:** Summary of Parameters Used in the Solid-State NMR Experiments

nucleus	^1^H	^13^C		^31^P	
sequence	^1^H SPE	^1^H–^13^C CP	^1^H–^13^C WISE	^31^P SPE	^1^H–^31^P CP
MAS rate	14 kHz	10 kHz	10 kHz	14 kHz	14 kHz
number of scans	32	1200	128	1200	1200
number of points	40,000	1024	along F1—32	2048	8192
			along F2—1024		
contact time		500 μs	500 μs		500 μs
recycle delay	5 s	5 s	5 s	10 or 50 s	5 s
90° pulse length	3 μs	3.6 μs (^1^H)	3.6 μs	4 μs	3.6 μs (^1^H)
spectral window	100 kHz	50 kHz	F1—100 kHz	50 kHz	50 kHz
			F2—50 kHz		

Fourier transform of the free induction decays was undertaken, using zero filling twice and exponential line broadening of 30, 50 and 50 Hz for ^1^H, ^13^C and ^31^P NMR spectra, respectively. The 2D ^1^H–^13^C WISE spectra were obtained similarly, using zero filling twice in both dimensions and exponential line broadening of 30 along F1 and 50 Hz along F2 dimension. The ^31^P chemical shifts were externally referenced to an 85% H_3_PO_4_ solution, using ammonium dihydrogen phosphate as the secondary reference; the ^1^H and ^13^C chemical shifts were externally referenced to tetramethylsilane, using adamantane and hexamethylbenzene as secondary references, respectively.

### 4.3 Water Extraction Procedure

To elucidate how the water-soluble P species present in PL and in some representative hydrochar samples were related to the ^31^P CP/MAS and SPE/MAS NMR spectra, a water extraction procedure was performed, following the same steps previously employed by Ghanim et al.:^32^ 0.5 g of the analyzed sample was shaken for 24 h in an orbital shaker with 40 mL of distilled water at room temperature and 200 rpm. The suspension was then centrifuged at 4000*g* for 10 min and filtered with a 0.45 μm filter paper; next, the solid residue of the extraction was dried overnight at 100 °C. As done with the original samples, ^31^P CP/MAS and SPE/MAS NMR experiments were conducted with the leached samples to compare the spectra obtained before and after water extraction.

### 4.4 XRD

XRD experiments were recorded for powdered samples using a Shimadzu XRD-6000 powder diffractometer with Cu-Kα radiation (λ = 1.5418 Å).

## Supporting Information Available

^31^P SPE/MAS NMR spectra of hydrochars produced with different initial pH in the presence of SA and AA; XRD of representative hydrochar samples (PDF)
