# Supplementary material for: Solid-State NMR Study of Hydrochars Produced from Hydrothermal Carbonization of Poultry Litter
Source: ACS Omega. 2024 Nov 4;9(46):45759–73. doi: 10.1021/acsomega.4c02876 (PMC11579733; doi:10.1021/acsomega.4c02876)
Supplement: Supplementary file 1 — ao4c02876_si_001.pdf [file ao4c02876_si_001.pdf]

SUPPORTING INFORMATION

Solid-state NMR study of hydrochars produced from hydrothermal carbonization of poultry litter

Mariana C. Santoro [1]*, Bashir M. Ghanim [2], Witold Kwapinski [3], James J. Leahy [3], Jair C. C. Freitas [1]

[1] Laboratory of Carbon and Ceramic Materials, Department of Physics, Federal University of Espírito Santo (UFES), Av. Fernando Ferrari, 514, 29075-910, Vitória, ES, Brazil.

[2] Department of Chemistry, The Higher Institute of Medical and Technical Sciences, 00000, Alzahra, Tripoli, Libya.

[3] Department of Chemical Sciences, Bernal Institute, University of Limerick, V94 T9PX Limerick, Ireland.

* Corresponding author: marianacsantoro@gmail.com

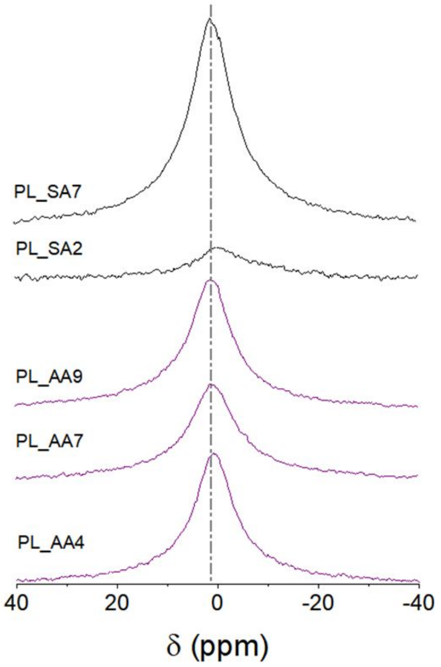

Figure S1. ^{31}P SPE/MAS NMR of hydrochars produced with different initial pH in the presence of sulfuric acid (SA) and acetic acid (AA).

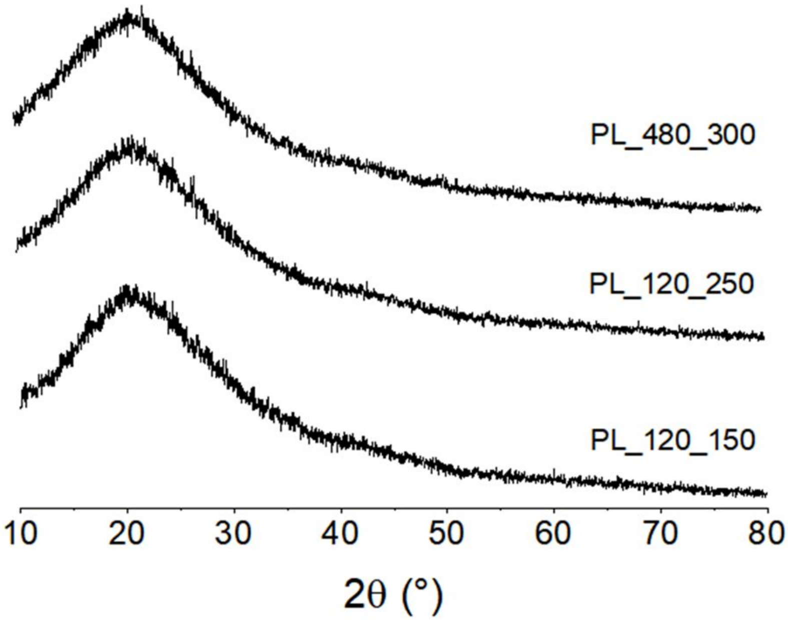

Figure S2. XRD patterns recorded at room temperature for HCs synthesized at different temperatures and residence times.
